# Comprehensive Multi-Omics Identification of Interferon-γ Response Characteristics Reveals That RBCK1 Regulates the Immunosuppressive Microenvironment of Renal Cell Carcinoma

**DOI:** 10.3389/fimmu.2021.734646

**Published:** 2021-11-02

**Authors:** Wenhao Xu, Juli Tao, Wenkai Zhu, Wangrui Liu, Aihetaimujiang Anwaier, Xi Tian, Jiaqi Su, Guohai Shi, Haineng Huang, Gaomeng Wei, Chuanyu Li, Yuanyuan Qu, Hailiang Zhang, Dingwei Ye

**Affiliations:** ^1^Department of Urology, Fudan University Shanghai Cancer Center, Shanghai, China; ^2^Department of Hematology and Rheumatology, Affiliated Hospital of Youjiang Medical University for Nationalities, Baise, China; ^3^Department of Neurosurgery, Affiliated Hospital of Youjiang Medical University for Nationalities, Baise, China; ^4^Department of Urology, Affiliated Hospital of Youjiang Medical University for Nationalities, Baise, China

**Keywords:** renal cell carcinoma, ccRCC kidney cancer, IFN-γ, immune checkpoint therapies, tumor microenvironment, RBCK1, bioinformatics, machine learning algorithm

## Abstract

Interferon-gamma (IFN-γ) has a complex role in modulating the tumor microenvironment (TME) during renal cell carcinoma (RCC) development. To define the role of IFN-γ response genes in RCC progression, we characterized the differential gene expression, prognostic implications, and DNA variation profiles of selected IFN-γ response signatures, which exhibited a significant hazard ratio for the overall survival (OS) and progression-free survival (PFS) of papillary, chromophobia, and clear cell RCC (ccRCC) patients (*n* = 944). Prognostic nomograms were constructed to predict the outcomes for ccRCC patients, highlighting the prognostic implications of RANBP2-type and C3HC4-type zinc finger containing 1 (RBCK1). Interestingly, large-scale pan-cancer samples (*n* = 12,521) and three single-cell RNA datasets revealed that RBCK1 showed markedly differential expression between cancer and normal tissues and significantly correlated with tumor-infiltrating immune cells, tumor purity, and immune checkpoint molecules, such as PD-L1, CTLA-4, LAG-3, and TIGIT in pan-cancer samples. Notably, the TIDE score was significantly higher in the RBCK1^high^ group compared with the RBCK1^low^ group in both ccRCC and RCC cohorts. Besides, immunohistochemistry staining showed significantly elevated RBCK1 expression in tumors (*n* = 50) compared with kidney samples (*n* = 40) from a real-world cohort, Fudan University Shanghai Cancer Center (FUSCC, Shanghai). After RBCK1 expression was confirmed in ccRCC, we found a significantly decreased number of infiltrating CD4^+^ T cells, CD4^+^ FOXP3^+^ Treg cells, M1 macrophages, and CD56^bight/dim^ NK cells in the immune-cold RBCK1^high^ group. In addition to the distinct heterogeneous immune microenvironment, the increased expression of RBCK1 predicted a prominently worse prognosis than the RBCK1^low^ group for 232 ccRCC patients in the FUSCC proteomic cohort. Furthermore, after transfected with siRNA in human ccRCC cells, extraordinarily decreased cell proliferation, migration capacities, and prominently elevated apoptosis tumor cell proportion were found in the siRNA groups compared with the negative control group. In conclusion, this study identified IFN-γ response clusters, which might be used to improve the prognostic accuracy of immune contexture in the ccRCC microenvironment. Immune-cold RBCK1^high^ patients have pro-tumorigenic immune infiltration and significantly worse outcomes than RBCK1^low^ patients based on results from multi-omics to real-world data. Our discovery of novel independent prognostic indicators for RCC highlights the association between tumor alterations and immune phenotype.

## Introduction

Although the diagnosis and treatment of renal cell carcinoma (RCC) have improved over the past 20 years, RCC is still one of the most fatal urinary malignancies. There were approximately 400,000 new RCC cases and 175,000 estimated deaths caused by RCC worldwide in 2018 (1). RCC can be classified as papillary RCC (pRCC), chromophobia RCC (chRCC), or clear cell RCC (ccRCC), which arise from the renal cortex or renal tubular epithelial cells and accounts for approximately 85% of all primary renal cancers (2).

Interferon (IFN) therapy, the first effective immunotherapy, has an important role in the treatment of RCC. In the era of targeted therapy, IFN is used to treat high-risk metastatic renal cell carcinoma patients in Japan and improve their survival (3). RCC patients with lung metastasis can benefit from combination therapy with IFN-α and IL-2 (4, 5). Currently, there are many strategies for the treatment of RCC including targeted therapies such as sorafenib and sunitinib or immunotherapies such as PD-L1 or PD-1 inhibitors. These new treatment regimens have important implications for the management of RCC. However, RCC is a highly heterogeneous cancer that lacks effective markers to guide prognosis and treatment (6). Furthermore, activation of tumor cell pro-cancer signals not only affects its own malignant biological behavior to promote tumor occurrence and development but also changes the metabolism and secretion of tumor cells and the tumor microenvironment, which alters the functional phenotype of tumor-infiltrating immune cells that aid in tumor immune escape (7, 8).

IFN-γ-signaling-related genes have an important role in the prognosis of RCC. Some IFN-γ-signaling-related genes, such as the IRF family genes, regulate the cell cycle and induce apoptosis in RCC cells (9). *RBCK1*, an IFN-γ-signaling-related gene, promoted p53 degradation *via* ubiquitination in RCC. The signature of IFN-γ-signaling-related genes may provide strategies to identify cancer-specific diagnosis and prognosis (10). A recent study found that PD-L1 was induced in ccRCC-like cell lines by canonical IFN-γ signaling and high levels of PD-L1 mRNA in tumor tissues were positively correlated with an IFN-γ signature that favorably affected prognosis (11). These findings suggest that interferon-related genes can be used to guide the treatment of immune checkpoint inhibitors.

The rapid development of bioinformatics has improved disease diagnosis, prognosis, treatment, phenotypes, and human phenomics (12–14). Different machine learning algorithms require rigorous statistics and real-world *in vitro* or clinical verification to determine tumor heterogeneity and the tumor microenvironment (15). This study hypothesized that IFN-related genes can be used to monitor the progression and long-term prognosis of renal cell carcinoma including clear cell RCC, chromophobe RCC, and papillary RCC and that these key genes can be used as simple biomarkers in a prediction model to predict the tumor-suppressive immune microenvironment and determine the poor prognosis of renal cell carcinoma.

## Methods

### Ethical Approval and Consent to Participate

All of the study designs and test procedures were performed in accordance with the Helsinki Declaration II. Ethical approval and participation consent of this study was reviewed and agreed upon by the Fudan University Shanghai Cancer Center (FUSCC) Institutional Review Board (No. 2008222-Exp49).

### Data Download and Sample Collection

The RNA-seq, copy number variation, mutation, and clinical and survival data of 530 ccRCC, 323 pRCC, and 91 chRCC patients were obtained from The Cancer Genome Atlas (TCGA, https://portal.gdc.cancer.gov) database with gene IDs converted from Ensembl ID to gene symbol matrix. The phenotypic and clinical data of large-scale pan-cancer samples (*n* = 12,521) were enrolled from the TCGA database. In addition, 232 ccRCC patients with proteomics sequencing and clinical data and 50 ccRCC together with 40 normal kidney FFPE samples were enrolled from the FUSCC cohort. The clinicopathological characteristics of 765 ccRCC patients from the TCGA and FUSCC cohort are described in **Supplementary Table 1**.

### Clinicopathological Characteristics and Survival Analysis

Student’s *t*-test was used to compare the expression levels of targeted genes in tumor and normal samples. Association between gene expression and clinicopathological characteristics was evaluated using Wilcoxon rank sum test and visualized using ggplot2 R package. The Kaplan–Meier survival with log-rank test was implemented to compare the survival benefit difference between groups. *p*-values and hazard ratio (HR) with 95% confidence interval (95% CI) were obtained using log-rank tests and univariate Cox proportional hazards regression. All the above analysis methods and R packages (ggrisk, survival, survminer, timeROC) were implemented by R Foundation for Statistical Computing version 4.0.3.

### DNA Variation Distribution Analysis

We downloaded the single nucleotide variation (SNV), copy number variation (CNV), genetic mutation data, transcriptome data, and clinical data from the TCGA database. To identify the somatic mutations of the patients with ccRCC, pRCC, and chRCC, mutation data were downloaded and visualized using the “maftools” package in R software. The profiles of SNV, CNV, and heterozygous CNV and the survival difference between CNV and the wide type of the IFN-γ response genes in RCC were analyzed based on Gene Set Cancer Analysis (GSCA, http://bioinfo.life.hust.edu.cn/GSCA), an integrated database for genomic and immunogenomic gene set cancer analysis.

### Construction of IFN-γ Response Gene Prediction Models and Nomogram

The least absolute shrinkage and selection operator (LASSO) Cox regression algorithm was used for the selection of independent indicators with 10-fold cross-validation using the R software package glmnet. The IFN-γ response gene prediction models (IPMs) for prognosis were constructed based on derived hub genes and corresponding assignment value. Besides, univariate and multivariate Cox regression analyses were performed to construct the nomogram enrolling IFN-γ response genes with the most prognostic implications and clinical features, such as age, pathological TNM stage, and ISUP grade using forestplot R package. The nomogram was developed based on the results of multivariate Cox proportional hazards analysis to predict the 1-, 3-, and 5-year survival recurrence.

### Assessment of Immune Cell Infiltration and Immune Checkpoint Expression

To develop reliable immune cell infiltration calculations, we implemented the immunedeconv R package which integrates xCell and CIBERSORT algorithms. Siglec-15, IDO-1, CD274, HAVCR2, PDCD1, CTLA-4, LAG-3, and PDCD1LG2 were selected to be immune checkpoints, and the expression level was extracted in pan-cancers. The association between target gene expression and immune cell infiltration of immune checkpoint expression level was assessed using Spearman’s test.

### Evaluation of Immunotherapy Efficacy in Patients With RCC

The Tumor Immune Dysfunction and Exclusion (TIDE) algorithm based on expression profile data has been used to predict the responsiveness of ccRCC and RCC patients receiving immune checkpoint inhibitors (ICIs) (16). Differentially expressed immune checkpoints between tumor groups and normal samples were assessed.

### Immunohistochemistry Staining and Tumor Immune Microenvironment Characterizations of ccRCC

Immunohistochemistry (IHC) was utilized to evaluate the expression level of RBCK1 (ab219955; Abcam) in 50 ccRCC and 40 adjacent normal tissues from the FUSCC Tissue Bank according to procedures previously described (17). Then, opal multispectral imaging was used to identify differential immune cell infiltration. CD3 (Kit-0003, Maxim, China), CD4 (RMA-0620, Maxim, China), CD8 (RMA-0514, Maxim, China), FOXP3 (98377, CST), CD20 (MAB-0669, Maxim, China), CD56 (99746, CST), CD68 (76437, CST), CD163 (MAB-0206, Maxim, China), PD-1 (84651, CST), and PD-L1 (13684, CST) antibodies were added to the slide and incubated overnight in a humidified chamber at 4°C. HRP-labeled goat anti-rabbit/mouse secondary antibody was added dropwise and incubated at room temperature. Finally, the slices are imaged and quantitatively analyzed on a multispectral imaging system (Vectra^®^ Polaris™, Shanghai).

### Assessment of RBCK1 Expression in Single-Cell RNA-Seq Datasets

Tumor Immune Single-Cell Hub (TISCH, http://tisch.comp-genomics.org/home/) was implemented to screen for scRNA-seq datasets with detailed cell-type annotation at the single-cell expression focusing on the tumor microenvironment in cancers. GSE111360 (*n* = 2, number of cells = 23,130), GSE139555 (*n* = 3, number of cells = 49,907), and GSE145281_PDL1 (*n* = 4, number of cells = 44,220) were included with correlation analyzed between RBCK1 expression and the abundance of immune cell infiltrations.

### Human ccRCC Cell Culture and Cell Transfection

The human ccRCC cell line, 786-O, was obtained from the National Collection of Authenticated Cell Cultures of China (No. TCHu186). The 786-O cells were cultured in culture medium RPMI-1640 (C11875500BT, GIBCO by Life Technologies, USA), supplemented with 10% fetal bovine serum (Hyclone, Life Sciences, Shanghai, China). Cells were incubated in a humidified atmosphere incubator of 5% CO_2_ at 37°C. 786-O cells have been transfected with double-stranded siRNA according to the protocol of the manufacturer using plasmid using Lipofectamine 3000 reagent (RiboBio) in a six-well plate, as previously described (17). The transfection dose for each well was 15 μl of siRNA1 (sequences: GGTGCACCTTCATCAACAA), siRNA2 (sequences: GGATTACCAGCGATTTCTA), or non-targeting siRNA (negative control) at a concentration of 10 nmol/L after incubated using RPMI-1640 for 20 min. 786-O cells were harvested for at least 24 h after transfection for further experimental analysis.

### Total mRNA Extraction and Real-Time Quantitative PCR

Total RNA sequence was isolated using TRIzol reagent (Invitrogen, Carlsbad, CA) from transfected and control cells. SYBR^®^ Premix Ex Taq™ (TaKaRa) was used to perform qRT-PCR reactions in triplicate according to the protocols of the manufacturer. The primers of RBCK1 were as follows: forward—5′-GCA GAT GAA CTG CAA GGA GTA TCA-3′ and reverse—5′-TGC AGC ATC ACC TTC AGC AT-3′. The relative RBCK1 expression quantity was measured after the 2^−ΔΔCt^ calculation using beta-actin as the internal standard.

### Cell Counting Kit-8 Assay

For viability assays, the transfected 786-O cells were seeded into 96-well plates at a density of 2,000 cells/well. Next, a 10-μl Cell Counting Kit (CCK)-8 solution (KeyGen Biotech, Nanjing, China) was added to each well, and cells were incubated at 37°C for an additional 2 h. The absorbance of each well at 450 nm was measured at 0, 1, 2, 3, 4, and 5 days after inoculation using an automatic microplate reader (Tecan, Switzerland) at 490 nm wavelength optical density. Three replicate analyses were performed for each sample.

### Flow Cytometry Assays for Cell Apoptosis

Apoptosis detection assay was performed using Annexin V-FITC Apoptosis Detection Kits (BD, USA) in accordance with the procedures of the manufacturer. Propidium iodide (PI) is a fluorescent dye that stains DNA and RNA and is used to analyze cell cycle by flow cytometry. Briefly, 786-O cells were seeded, triple washed with PBS, and then treated with 5 μl Annexin V-FITC and 5 μl PI solution in each collection tube. After incubation for 15 min, cell apoptosis was analyzed using a FACS analyzer (BD, USA).

### Transwell Migration Assay

The transfected 786-O cells were seeded in a medium with 10% FBS and placed in each Transwell chamber at a density of 1 × 10^5^ cells/well. The medium containing 20% fetal bovine serum was added in the lower 24-well plate chamber. After 24 h, the bottom 786-O cells were treated with 4% polyoxymethylene for 15 min and 0.1% crystal violet for 30 min. Then, 786-O cells migrating to the lower surface of the Transwell chamber were counted using a microscope in six random fields.

### Statistical Analysis

SPSS 20.0, GraphPad Prism 8.0, and R software were used for all statistical analyses. The R packages used are listed in the corresponding subheading of the *Methods* section. All statistical analyses were two-sided, and a value of *p <*0.05 was considered statistically significant.

## Results

### IFN-γ Response Genes Are Differentially Expressed Between RCC and Normal Kidney Tissues

**Figure 1:**
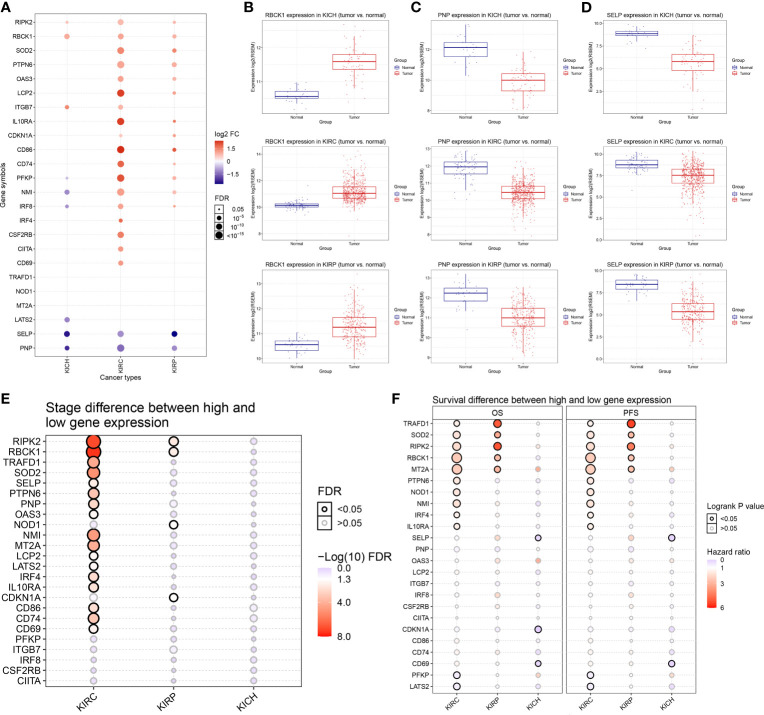
Differentially expressed 24 IFN-γ response genes and its association with clinical features of renal cell carcinoma (RCC). **(A)** We compared the mRNA expression in clear cell RCC (ccRCC) (*n* = 530), papillary RCC (pRCC) (*n* = 323), chromophobia RCC (chRCC) (*n* = 91), and adjacent normal kidney tissues displayed using a bubble chart. The size of the circle represents statistical significance; red represents significantly high expression in tumor tissue and blue represents low. **(B–D)** Expressions of RBCK1, PNP, and SELP in RCC samples were observed in comparison with normal samples using unpaired *t*-test. **(E)** We summarized the difference of IFN-γ response signatures of mRNA expression between clinical stages in ccRCC, pRCC, and chRCC using Wilcoxon non-parametric test. **(F)** The univariate Cox survival analysis emphasized the prognostic significance of TRAFD1, SOD2, RIPK2, RBCK1, and MT2A as cancer-promoting factors of ccRCC and pRCC.

To assess the characteristics of IFN-γ response genes in RCC, we compared the mRNA expression in ccRCC (*n* = 530), pRCC (*n* = 323), chRCC (*n* = 91), and adjacent normal kidney tissues (*n* = 128). The differentially expressed mRNAs are shown in a bubble chart in **Figure 1A**. The size of the circle represents statistical significance—red and blue represent significantly high or low expression in tumor tissues, respectively. Notably, most IFN-γ response genes showed markedly differential expression patterns, although some IFN-γ response genes had similar expression patterns between the three kidney cancer subtypes. For example, the expression of RBCK1 was significantly higher in RCC tumors compared with normal samples (**Figure 1B**). Significantly decreased PNP and SELP expressions in RCC samples were observed when compared with normal samples (**Figures 1C, D**).

### Association Between IFN-γ Response Genes and the Clinical Features of RCC

Next, we summarized the differences in IFN-γ response mRNA signatures between different clinical stages of ccRCC, pRCC, and chRCC (**Figure 1E**). The expressions of RIPK2, RBCK1, TRAFD1, SOD2, NMI, MT2A, CD86, and CD74 were significantly increased in the advanced stages of ccRCC, and the expressions of RIPK2, RBCK1, NOD1, and CDKN1A were significantly increased in the advanced stages of pRCC. In addition, the survival analysis emphasized the prognostic significance of TRAFD1, SOD2, RIPK2, RBCK1, and MT2A as cancer-promoting factors in ccRCC and pRCC (**Figure 1F**). The survival curves of IFN-γ response signatures with significant prognostic value for ccRCC (**Supplementary Figure 1**) and pRCC patients (**Supplementary Figure 2**) are displayed. Taken together, we identified 24 IFN-γ response signatures with significantly different expressions that had strong prognostic implications among 947 RCC samples.

### DNA Variations in 24 IFN-γ Response Signatures in RCC

**Figure 2:**
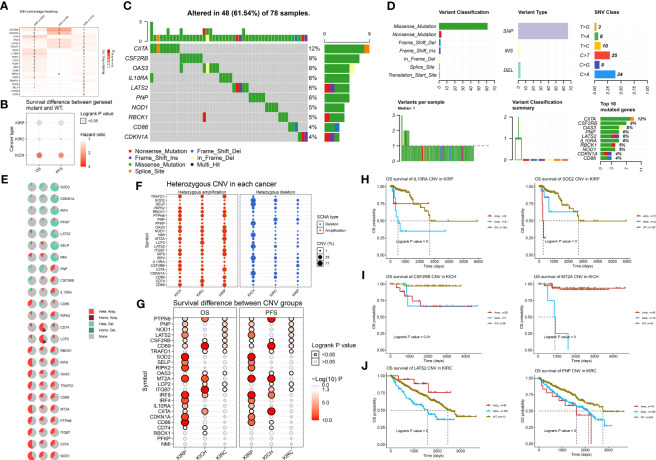
DNA variation landscape of 24 IFN-γ response signatures of RCC. **(A)** Profiles of SNV of the 24 IFN-γ response signatures in ccRCC (*n* = 370), pRCC (*n* = 282), and chRCC (*n* = 66) patients were provided. **(B)** Mutation of IFN-γ response signatures was used to predict poor survival for patients with RCC. **(C)** Mutation information of IFN-γ response signatures in 78 RCC samples with DNA variation was shown in the waterfall plot, where different colors with specific annotations at the bottom meant various mutation types. **(D)** The variant classifications, variant type, and SNV classes of IFN-γ response signatures were summarized. A stacked bar plot shows the top 10 mutated genes. **(E)** Pie plot summarizes the CNV of IFN-γ response signatures in patients with ccRCC, pRCC, and chRCC. **(F)** Using bubbles to represent the percentage of heterozygous CNV, including heterozygous amplification and deletion of IFN-γ response signatures in ccRCC, pRCC, and chRCC. The bubble size is positively correlated with percentage; the bubble color blue represents deletion, and red represents amplification. **(G)** The hazard ratio and log-rank *p*-value through bubble color and size. The column is the gene set symbol and the row is the ccRCC, pRCC, and chRCC. The bubble color from blue to red represents the significance of log-rank *p*-value from low to high, and bubble size is positively correlated with the significance of log-rank *p*-value. **(H–J)** Kaplan–Meier curves between amplification, deletion, and wide type of the 24 IFN-γ response signatures in ccRCC, pRCC, and chRCC.

Next, we examined DNA variation in the IFN-γ response signatures of RCC. As shown in **Figure 2A**, we investigated the profiles of SNV of the 24 IFN-γ response signatures in ccRCC (*n* = 370), pRCC (*n* = 282), and chRCC (*n* = 66) patients. These findings indicate a high mutation frequency of *CIITA* and *LATS2* in ccRCC; *CIITA*, *IL10RA*, and *OAS3* in pRCC; and *CSF2RB* and *CDKN1A* in chRCC. Besides, although statistical significance was not observed for gene expression or survival in the chRCC dataset, mutations in the IFN-γ response signatures predicted relatively poor survival for patients with chRCC (**Figure 2B**). Next, we examined the SNV of the top 10 mutated genes among IFN-γ response signatures. The most frequently mutated genes were *CIITA*, *CSF2RB*, and *OAS3* in 78 RCC samples with DNA variation (**Figure 2C**). The variant classifications, variant type, and SNV classes of IFN-γ response signatures are summarized in **Figure 2D**.

### Relationship Between the CNV of the 24 IFN-γ Response Signatures and the Clinical Outcomes of RCC

After determining the DNA mutation map, we determined whether the CNV was characteristic of IFN-γ response signatures in kidney cancer. **Figure 2E** shows the CNV characteristics of IFN-γ response signatures in patients with ccRCC, pRCC, and chRCC. The CNV of SOD2, CDKN1A, and IRF4 was mainly reflected in Hete.Del, and the CNV of CD74 and LCP2RBCK1 was mainly reflected in Hete.Amp. Next, we explored the heterozygous amplification and deletion of IFN-γ response characteristics (**Figure 2F**). There was a high percentage of heterozygous CNV of IFN-γ response signatures in ccRCC, pRCC, and chRCC patients. Next, we explored the prognosis implications between CNV groups and a wild type (WT) group using the Kaplan–Meier method (**Figure 2G**). The results showed that CNV changes in many interferon-related genes showed inconsistent survival interventions for RCC patients compared with the WT group (*p *< 0.05, log|FC| > 1). Next, we showed that interferon genes had a high frequency of CNV among the three kidney cancer subtypes and significantly interfered with the overall survival prognosis. For example, the CNV of IL10RA and SOD2 indicated a significantly worse overall survival (OS) for pRCC patients compared with the WT group. For chRCC patients, the CNV of CSF2RB indicated a significantly worse OS, and patients with a deletion of MT2A had poor outcomes compared with the WT and amplification groups. Interestingly, LATS2 had a higher CNV percentage in ccRCC SCNA deletions, whereas its amplification and deletion had significantly opposite prognostic implications compared with the WT group; the CNV of PNP indicated a significantly worse OS for ccRCC patients compared with the WT group (**Figures 2H**–**J**). Overall, we found a strong relationship between the CNV profiles of the 24 IFN-γ response signatures and the clinical outcomes of ccRCC, pRCC, and chRCC patients.

### IPMs Significantly Predict Prognosis and Immune Cell Infiltration of ccRCC

**Figure 3:**
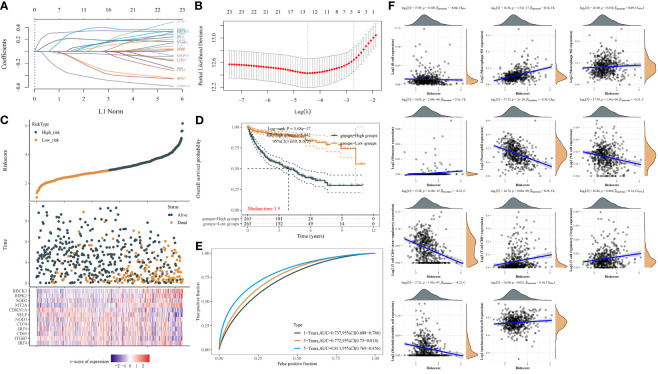
IFN-γ response signature prediction models (IPMs) predicting progression-free survival (PFS) and immune cell infiltration of ccRCC. **(A)** Coefficients of the 24 IFN-γ response signatures are displayed by the lambda parameter. **(B)** Partial likelihood deviance *versus* log (*λ*) was drawn using LASSO Cox regression model. **(C)** The dotted line represented the median risk score and divided the patients into low-risk and high-risk groups. **(D)** Risk score of the 24 IPMs for predicting PFS of ccRCC patients from TCGA was displayed using Kaplan–Meier curves. *p*-values and hazard ratio (HR) with 95% confidence interval (CI) were generated by log-rank tests and univariate Cox proportional hazards regression. **(E)** Time-dependent ROC analysis of the IPMs was generated in predicting 1-, 3-, and 5-year progression status. **(F)** Spearman correlation analysis of IPMs and immune cell infiltration. The horizontal axis in the figure represents the expression distribution of risk score of IPMs, and the ordinate is the expression abundance of immune cell infiltration.

Next, we used the LASSO regression model to derive risk characteristics of the IFN-γ response signatures for progression-free survival (PFS) and OS in ccRCC and all RCC patients (**Figures 3A, B**). Based on the median risk score, patients were divided into low- and high-risk groups. We found that the risk score increased with increased mortality events (**Figure 3C**). Next, the risk score of the 24 IPMs to predict the PFS of ccRCC patients from the TCGA was calculated (**Figure 3D**). We found that an elevated risk score of IPMs was significantly correlated with a worse prognosis for ccRCC patients (HR = 5.442, *p *< 0.001). We also conducted ROC analysis on IPMs and found AUC values with strong specificity and sensitivity (1-year = 0.737, 3-year = 0.772, 5-year = 0.813), which indicated that it is an effective and accurate marker for predicting the PFS of ccRCC patients (**Figure 3E**). In addition, we analyzed the relationship between IPMs and the amount of immune cell infiltration. The analysis demonstrated that the risk score of IPMs was significantly correlated with the upregulation of M1 macrophages and the downregulation of CD4^+^ T cells, neutrophils, and NK cells (Spearman’s correlation analysis, *p *< 0.001) (**Figure 3F**).

**Figure 4:**
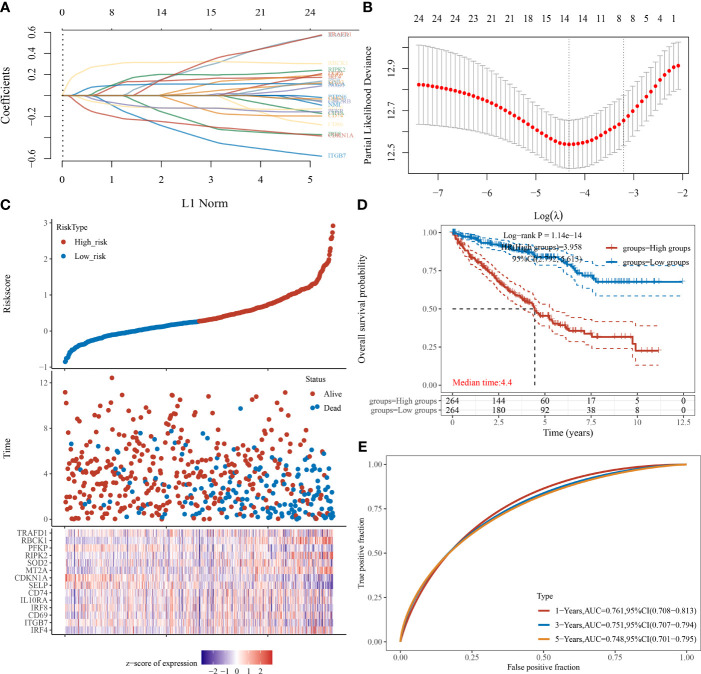
IPMs predicting the OS of ccRCC. **(A)** Coefficients of the 24 IFN-γ response signatures are displayed by the lambda parameter. **(B)** Partial likelihood deviance *versus* log (*λ*) was drawn using LASSO Cox regression model. **(C)** The dotted line represented the median risk score and divided the patients into low-risk and high-risk groups. **(D)** Risk score of the 24 IPMs for predicting OS of ccRCC patients from TCGA was displayed using Kaplan–Meier curves. *p*-values and hazard ratio (HR) with 95% confidence interval (CI) were generated by log-rank tests and univariate Cox proportional hazards regression. **(E)** Time-dependent ROC analysis of the IPMs was generated predicting 1-, 3-, and 5-year survival status.

We also used the LASSO regression model to derive the risk characteristics of IFN-γ response genes to predict the OS of ccRCC patients (**Figures 4A, B**). Based on the median risk score of the IPMs, patients were divided into low- and high-risk groups (**Figure 4C**). **Figure 4D** shows that a higher risk score of IPMs predicted a significantly worse prognosis for ccRCC patients (HR = 3.958, *p *< 0.001). ROC analysis revealed high AUC values (1-year = 0.761, 3-year = 0.751, 5-year = 0.748) of the IPMs (**Figure 4E**). These findings indicate that IPM can be used as an independent prognostic predictor of ccRCC.

### IPMs Significantly Predict the PFS and OS of All RCC Patients

In addition, we used the LASSO regression model to derive the risk characteristics of IFN-γ response signatures for PFS and OS in all RCC patients (**Supplementary Figures 3A, B**). We found that the higher the risk score, the worse the prognosis of the RCC patient (HR = 4.31, *p *< 0.001), which suggests the prognostic independence of the IPMs to predict the PFS of RCC patients (1-year AUC = 0.821, 3-year AUC = 0.757, 5-year AUC = 0.758) (**Supplementary Figures 3C**–**E**). We then analyzed the relationship between IPMs and immune cell infiltration of RCC and found that the risk score of IPMs had a significant positive correlation with M1 macrophages and a negative correlation with CD4^+^ T-cell infiltration (*p *< 0.001, |*r*^2^| > 0.30) (**Supplementary Figure 3F**).

Then, we investigated the prognostic effect of IPMs to predict the OS for RCC patients (**Supplementary Figure 4**). We found that the higher the risk score, the worse the prognosis of the RCC patient (HR = 5.23, *p *< 0.001) with high AUC values (1-year = 0.790, 3-year = 0.769, 5-year = 0.788), which indicated that it is an effective and accurate model to predict the prognosis of ccRCC and all RCC patients.

### Identification of Differentially Expressed Genes of IPMs and Functional Annotations

**Figure 5:**
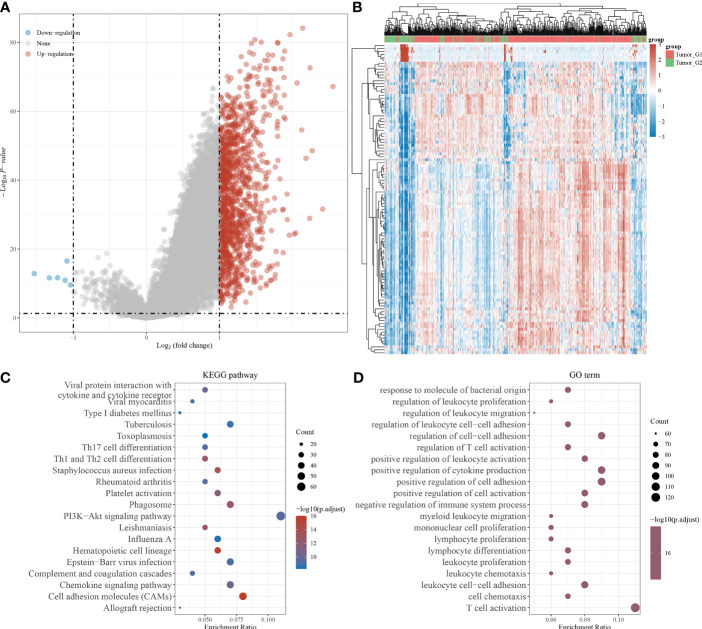
Identification of differentially expressed genes (DEGs) of IPMs and functional annotations. **(A)** Volcano plots were constructed using fold-change values and adjusted *p* based on the high and low risk score of IPMs for predicting PFS of ccRCC patients from TCGA. The red point in the plot represents the overexpressed mRNAs and the blue point indicates the downexpressed mRNAs with statistical significance. **(B)** Hierarchical clustering analysis to identify DEGs of IPMs in ccRCC, which were differentially expressed between tumor and normal tissues. **(C, D)** The enriched KEGG signaling pathways and GO functional enrichment (including biological process, cellular component, and molecular function) analysis were selected to demonstrate the primary biological actions of major DEGs of IPMs based on ClusterProfiler package in R software.

We screened differentially expressed genes (DEGs) related to IPMs and performed functional annotations to identify the potential role of IPMs in ccRCC patients. First, we screened related genes according to the high- and low-risk scores of IPMs (**Figure 5A**). Then, we determined the DEGs of IPMs in ccRCC *via* hierarchical cluster analysis and analyzed their differences between tumor and normal tissues (**Figure 5B**). Finally, we conducted a selective enrichment of the Kyoto Encyclopedia of Genes and Genomes (KEGG) signaling pathway and Gene Ontology (GO) functional enrichment analysis (including biological processes, cell components, and molecular functions) of the selected DEGs of IPMs to demonstrate their main biological effects (**Figures 5C, D**). In the enrichment of GO functions, DEGs were mainly related to viral protein interactions with cytokines and cytokine receptors, type I diabetes mellitus, and viral myocarditis. In the KEGG signaling pathway, DEGs were mainly related to responses to molecules of bacterial origin, the regulation of leukocyte proliferation, and the regulation of leukocyte migration.

### Construction of a Nomogram to Predict the Prognosis for ccRCC Patients

**Figure 6:**
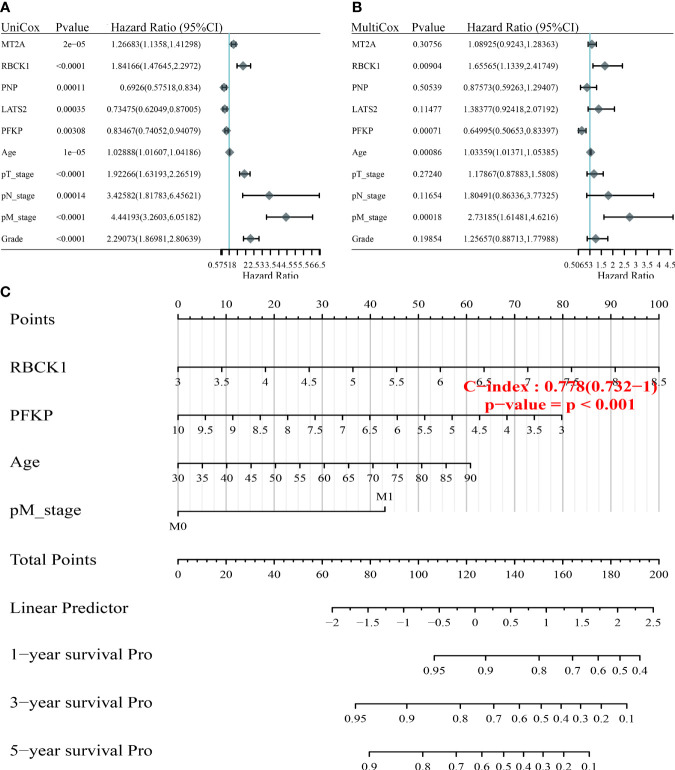
Construction of the nomogram predicting the prognosis of ccRCC. **(A, B)** Hazard ratio and *p*‐value of constituents involved in univariate and multivariate Cox regression enrolling clinicopathological features and five IFN-γ response signatures with the most prognostic significance. **(C)** The nomogram provided a graphical representation of the clinicopathological features and IFN-γ response signatures, which can be used to calculate the risk of recurrence for an individual patient by the points associated with each risk factor through the “rms” R package.

To optimize the predictive models and enhance clinical translation efficiency based on the prognostic implications of the 24 IFN-γ response signatures, we selected the five IFN-γ related genes (*MT2A*, *RBCK1*, *PNP*, *LATS2*, *PFKP*) with the most prognostic significance for ccRCC for use in a nomogram. Using univariate and multivariate Cox regression analyses, we found that RBCK1 was the most clinically significant factor to predict outcomes for ccRCC patients (*p *< 0.01) (**Figures 6A, B**). The constructed nomogram indicated the significant prognostic roles of RBCK1 and PFKP (C-index = 0.761) (**Figure 6C**). Then, we aimed to establish a nomogram to predict the PFS of ccRCC patients using univariate and multivariate Cox regression, and found that RBCK1 was the most clinically significant (*p *= 0.027) (**Supplementary Figures 5A, B**). The nomogram also indicated the significant prognostic role of RBCK1 (C-index = 0.761) for ccRCC patients (**Supplementary Figure 5C**).

### Protein–Protein Interaction Networks and the Single-Cell Localization of RBCK1

**Figure 7:**
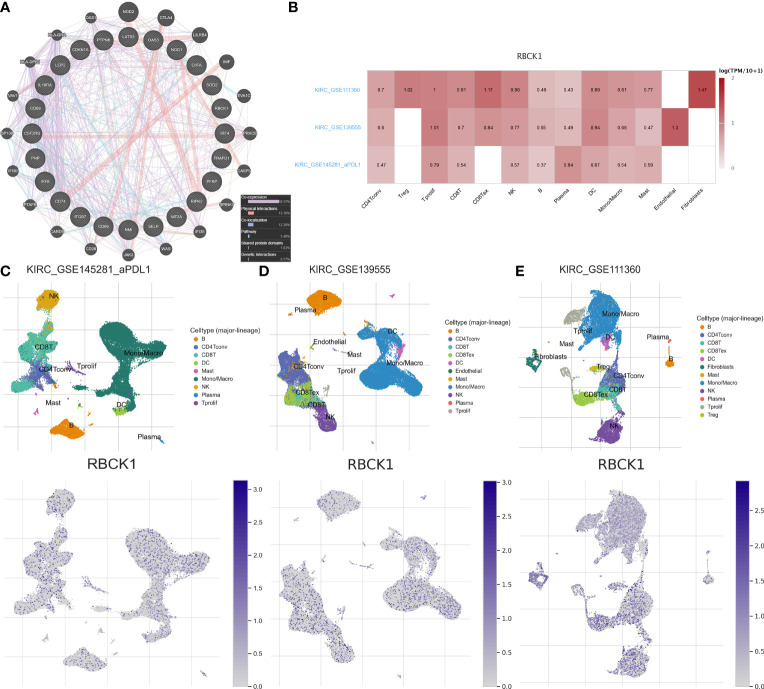
Protein networks and single-cell localization of RBCK1. **(A)** Protein–protein interaction (PPI) network of the 24 IFN-γ response signatures was constructed using GeneMANIA. **(B)** Three scRNA-seq datasets with detailed cell-type annotation at the single-cell level focusing on the tumor microenvironment for ccRCC. GSE111360 (*n* = 2, number of cells = 23,130), GSE139555 (*n* = 3, number of cells = 49,907), and GSE145281_PDL1 (*n* = 4, number of cells = 44,220) were enrolled with correlation analyzed between *RBCK1* expression and abundance of immune cell infiltrations. The higher the expression of *RBCK1* in a single-cell subpopulation, the darker the red color is in the heatmap. **(C–E)** The heatmap showed the relatively high expression of *RBCK1* in different cell types, such as CD^4+^, CD^8+^ T cells, monocytes, macrophages, and NK cells across the three scRNA-seq datasets.

After screening the most significant gene, *RBCK1*, we constructed a protein–protein interaction (PPI) network and performed single-cell localization analysis. We used GeneMANIA to construct a PPI network with the 24 IFN-γ response characteristics (**Figure 7A**). As shown in **Figure 7B**, RBCK1 was highly expressed in different cells in the tumor immune microenvironment (TIME), including CD4^+^ and CD8^+^ T cells, monocytes, macrophages, and NK cells. Using three scRNA-seq datasets, we detailed cell-type annotations at the single-cell level and focused on the tumor microenvironment of ccRCC to analyze the correlation between RBCK1 expression and immune abundance (**Figures 7C**–**E**). Overall, RBCK1 was widely expressed in a variety of cell types in the TIME of ccRCC and may mediate malignant clinical phenotypes by regulating the microenvironment.

### Pan-Cancer Analysis of RBCK1 mRNA Differential Expression and Correlation Analysis of TIME

**Figure 8:**
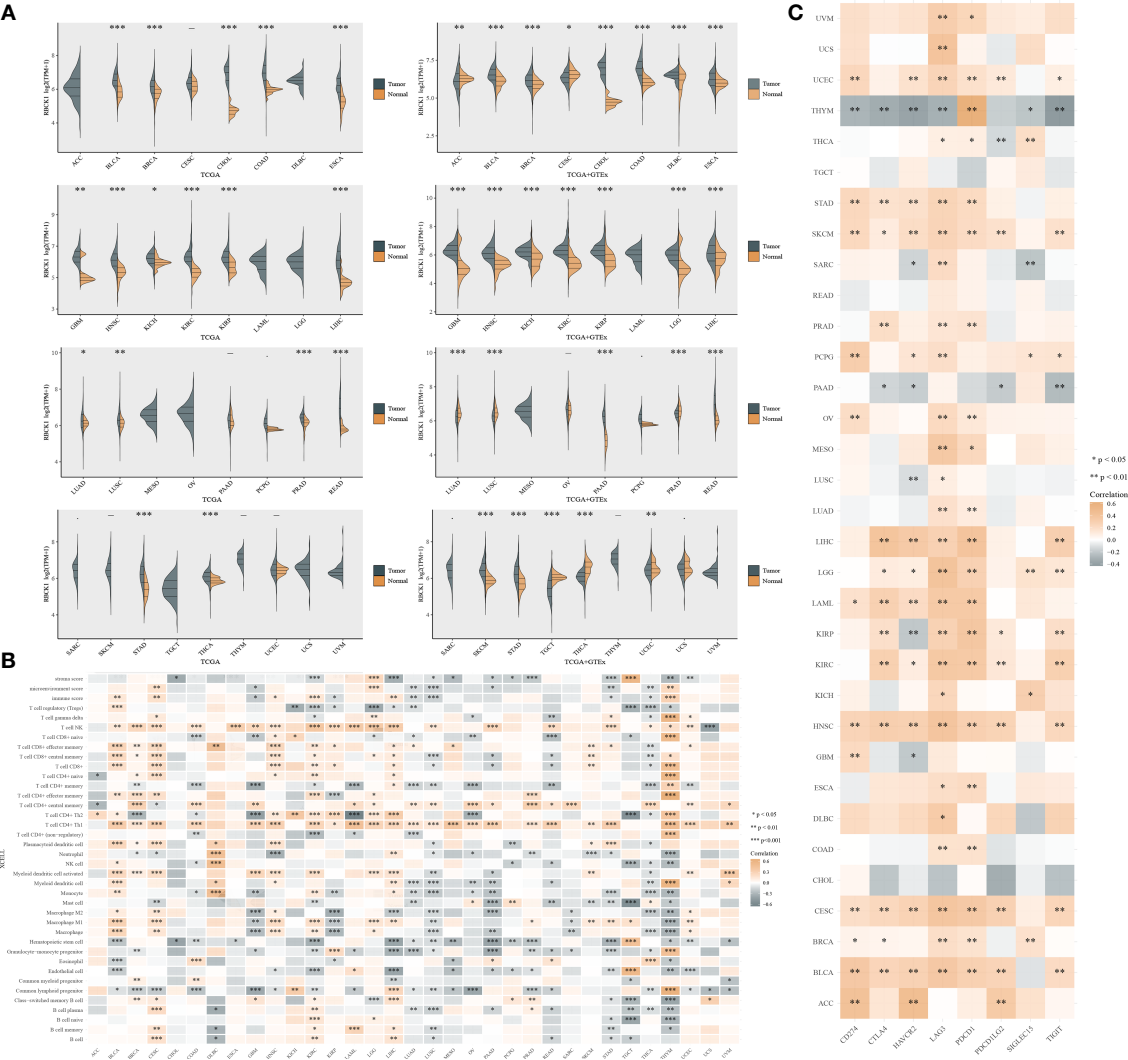
Pan-cancer analysis of RBCK1 mRNA differential expression and correlation analysis of the tumor immune microenvironment. **(A)** The expression distribution of RBCK1 in tumor and normal samples from the TCGA database and GTEx Portal, where the horizontal axis represents different tumor tissues, and the vertical axis represents the gene expression distribution using the Wilcoxon test (*p < 0.05, **p < 0.01, ***p < 0.001). **(B)** Spearman correlation analysis heatmap of immune score and RBCK1 expression in over 13,000 pan-cancer tissues, where the horizontal axis represents different tumor tissues, and the vertical axis represents different immune scores. The stronger the correlation, the darker the color. **(C)** The heatmap represents the correlation between RBCK1 expression and immune checkpoints (SIGLEC15, IDO1, CD274, HAVCR2, PDCD1, CTLA4, LAG3, and PDCD1LG2) in pan-cancer tissues, where the horizontal axis represents different immune checkpoint genes, and the vertical axis represents different tumor tissues (*p < 0.05, **p < 0.01, ***p < 0.001).

By single-cell analysis, we analyzed the expression of RBCK1 in pan-cancer tissues (**Figure 8A**). We analyzed the expression distribution of RBCK1 in tumor and normal samples from the TCGA database and GTEx Portal and found the significantly different expression of RBCK1 in pan-cancers, such as bladder cancer, breast cancer, glioma, lung cancer, and liver cancer. Next, we analyzed the enrichment score of all TIME elements and RBCK1 in pan-cancers and found that the expression of RBCK1 was significantly correlated with increased numbers of immune cells, including B cells, Th2 cells, M1 macrophages, and the decreased numbers of CD4^+^ T cells and Treg cells (**Figure 8B**). Moreover, we investigated the relationship between RBCK1 expression and immune checkpoint genes, including *SIGLEC15*, *IDO1*, *CD274*, *HAVCR2*, *PDCD1*, *CTLA4*, *LAG3*, and *PDCD1LG2* in pan-cancers. Interestingly, RBCK1 showed significant differential expression between cancer and normal tissues and was significantly related to tumor-infiltrating immune cells, including tumor purity and immune checkpoint molecules such as PD-L1, CTLA-4, LAG-3, and TIGIT in ccRCC (**Figure 8C**). Although RBCK1 expression was positively correlated with most cancer types, its expression had a significant negative association with immune checkpoints in thymoma samples.

### Immunotherapy Efficacy, TIME Characterization, and Prognostic Implications of RBCK1 in ccRCC Patients From the FUSCC Proteomics Cohort

**Figure 9:**
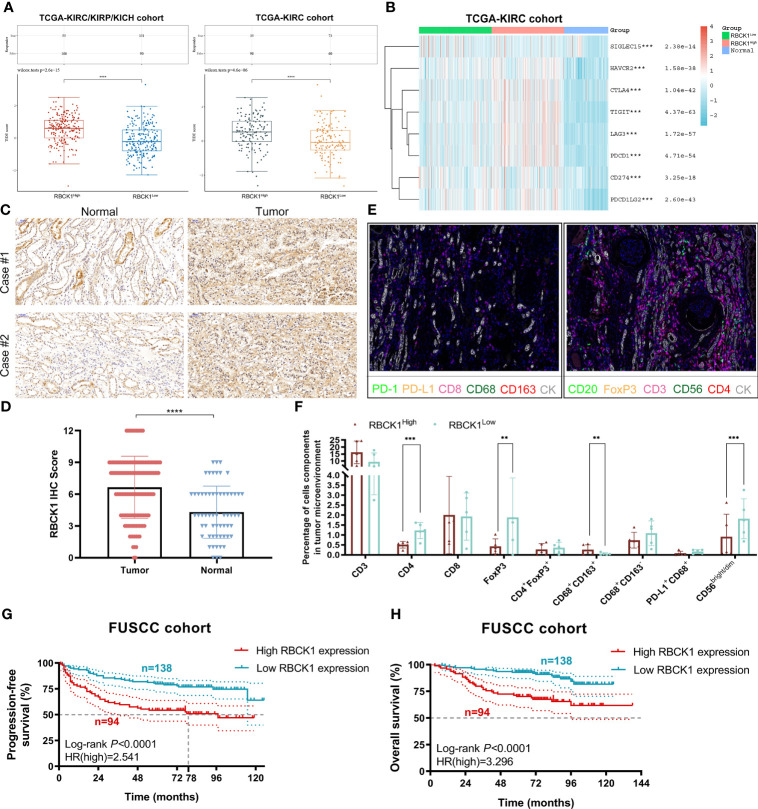
Immunotherapy efficacy, TIME characterization, and prognostic implications of RBCK1 in ccRCC patients from the Fudan University Shanghai Cancer Center (FUSCC) proteomics cohort. **(A)** The TIDE algorithm is used to evaluate two different tumor immune escape mechanisms and was developed in RBCK1^high^ compared with low RBCK1 expression group in both ccRCC and RCC cohorts using Student’s *t*-test. **(B)** The expression heatmap of immune checkpoint-related genes and RBCK1 in ccRCC and normal tissues was tested by Kruskal–Wallis, and the different colors represent the expression trend in different samples. **(C, D)** Immunohistochemistry staining showed RBCK1 expression in tumor (*n* = 50) and normal kidney samples (*n* = 40) from a real-world cohort, FUSCC, Shanghai. **(E, F)** Opal multi-marker immunohistochemistry was used on 10 ccRCC specimens to achieve six biomarker staining. The significance of the two groups of samples passed the Wilcoxon test. **(G, H)** Prognostic value of RBCK1 in predicting prognosis was assessed for 232 ccRCC patients from the FUSCC cohort based on proteomics sequencing data using the Kaplan–Meier method (**p < 0.01, ***p < 0.001, ****p < 0.0001).

The TIDE algorithm was used to evaluate two different tumor immune escape mechanisms, and was used to compare RBCK1^high^ and RBCK1^low^ expressing groups in the ccRCC or RCC cohorts (**Figures 9A, B**). The TIDE score was significantly higher in the RBCK1^high^ group compared with the RBCK1^low^ group in ccRCC and RCC patients, which suggests that RBCK1^high^ patients are intolerant to ICT treatment. Kruskal–Wallis analysis showed that immune checkpoints were significantly associated with the expression level of RBCK1 in ccRCC (**Figure 9B**). Besides, as shown in the revised **Supplementary Figure 6**, we enrolled a total of 136 patients with ccRCC from the RECA-EU cohort in the International Cancer Genome Consortium (ICGC) database. Consistent with TCGA database results, it suggested that TIDE score in ccRCC samples with elevated RBCK1 expression is significantly higher in samples with low RBCK1 expression (*p *= 2.5e-08). Immunohistochemistry staining showed significantly elevated RBCK1 expression in tumors (*n* = 50) compared with normal kidney samples (*n* = 40) from the FUSCC real-world cohort (*p *< 0.001; **Figures 9C, D**). After RBCK1 expression of ccRCC was confirmed, we found a significantly decreased number of infiltrated CD4^+^ T cells, CD4^+^FOXP3^+^ Treg cells, CD68^+^CD163^−^ M1 macrophages, and CD56^bight/dim^ NK cells in the immune-cold RBCK1^high^ group using opal multi-marker immunohistochemistry staining (**Figures 9E, F**). In addition to the heterogeneously distinct immune microenvironment, RBCK1^high^ predicted a significantly worse prognosis than RBCK1^low^ for 232 ccRCC patients in the FUSCC cohort based on proteomics sequencing data (PFS: HR = 2.541, *p *< 0.001; OS: HR = 3.296, *p *< 0.001; **Figures 9G, H**).

### Inhibition of RBCK1 Attenuates Cell Proliferation and Migration and Promotes Cell Apoptosis Abilities of 786-O Cells

**Figure 10:**
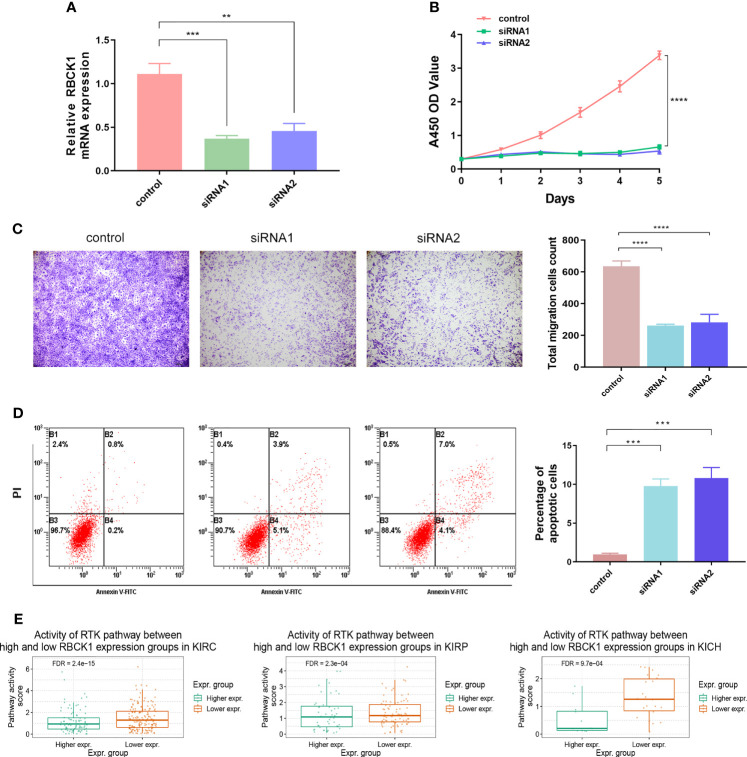
Inhibition of RBCK1 attenuates cell proliferation and migration and promotes cell apoptosis abilities of 786-O cell. **(A)** SiRNA was used to knock down the expression of RBCK1 in the human ccRCC cell line, 786-O. **(B, C)** The CCK-8 and Transwell assays were detected after the siRNAs were transfected in human ccRCC cells. **(D)** Early and late apoptotic cell proportions using the FITC/PI kit. Cell populations with FITC−/PI−, FITC+/PI−, FITC+/PI+, and FITC−/PI+ were regarded as living, early apoptotic, late apoptotic, and necrotic cells, respectively. **(E)** Differential RBCK1 inactivation level in the score of receptor tyrosine kinase (RTK) pathway in RCC samples from TCGA (**p < 0.01, ***p < 0.001, ****p < 0.0001).

To further investigate the biological function of the RBCK1 in ccRCC, we used siRNA to knock down the expression of RBCK1 in the human ccRCC cell line, 786-O. The relative expression of RBCK1 was significantly inhibited in the siRNA groups compared with the negative control group (*p *< 0.01; **Figure 10A**). Furthermore, after we transfected with siRNA in human ccRCC cells, the CCK-8 and Transwell assays revealed extraordinarily markedly decreased cell proliferation and migration capacities in the siRNA groups compared with the negative control group (*p *< 0.001; **Figures 10B, C**). In addition, we assessed early and late apoptotic cell proportions using the FITC/PI kit. Cell populations with FITC−/PI−, FITC+/PI−, FITC+/PI+, and FITC−/PI+ were regarded as living, early apoptotic, late apoptotic, and necrotic cells, respectively. The results suggested significantly increased apoptotic cell percentage of the siRNA groups compared with the control group (*p *< 0.001; **Figure 10D**). Interestingly, we then analyzed the downstream signaling pathway RBCK1 was possibly involved in and found that RBCK1 inactivation significantly promotes receptor tyrosine kinases (RTK) pathway in RCC samples from TCGA (*p *< 0.001; **Figure 10E**).

## Discussion

The incidence of RCC is increasing and it has become the most common kidney cancer. Over the past decade, various therapeutic options have been developed including cytoreductive nephrectomy, targeted therapies, and immune checkpoint inhibitors for advanced RCC patients (18). RCC has heterogeneous genomic and clinical features, and a better understanding of the molecular biology of tumors might help in the diagnosis and choice of treatment approaches. Therefore, many biomarkers associated with treatment outcomes and disease-specific outcomes have been identified. In addition, predictive signatures have been developed based on the multi-omics analysis of renal cell carcinoma. The most well-known hallmark of RCC, especially ccRCC, is VHL inactivation (19, 20). However, VHL inactivation alone is insufficient for the induction of RCC tumorigenesis. PBRM1, BAP1, SETD2, and PTEN mutations also commonly occur in RCC (21, 22). A risk model that included PBRM1, BAP1, and TP53 mutation status was correlated with the OS and PFS of RCC patients. PBRM1 mutations occur in almost 30%–40% of ccRCC tumors (23), and patients with PBRM1 loss have a poor predicted clinical outcome (22). The recent development of ICI treatment has shifted immunotherapy into the first-line setting and the combination of target therapies and ICIs is on the horizon, and a suitable predictive signature can direct the choice of combination therapies (24). In chRCC, tumor mutation burden (TMB) is associated with tumor metastasis and tumor grade. The immune-related genes *BIRC5*, *INHBE*, and *IL20RB* were upregulated in a TMB^high^ group and were associated with a poor prognosis (25), and a combination of PD-1 and CTLA-4 blockers was suggested for the treatment of advanced renal cell carcinoma with aberrations (26).

Cancer cells are the key responders of IFN-γ in the tumor microenvironment, and IFN-γ drives immune-activated and immune-suppressive effects (27). The immune activation of IFN-γ on tumor cells is largely attributed to tumor cells, monocytes, endothelial cells, and fibroblasts inducing tumor cells to express MHC class I and secrete CXCL9, CXCL10, and CXCL11 to promote lymphocyte migration and inhibition of angiogenesis (28–30). Therefore, it is worth exploring which cells expressing IFN-γ in the tumor microenvironment mediate the resistance of immunotherapy and the mechanism of IFN-γ-mediated “immune-cold” tumor turning to “immune-hot,” in order to design better treatment methods to balance antitumor and immune escape capacities of RCC (31, 32). In the present study, we examined the expression, prognostic implications, and copy number variation profiles of 24 IFN-γ response genes that were significantly correlated with OS and PFS for papillary, chromophobia, and clear cell RCC (*n* = 947). We derived a risk signature for use in a LASSO Cox regression model comprised of the IFN-γ response genes for prognosis. We found a marked upregulation of M1 macrophages and downregulation of CD4^+^ T cells, neutrophils, and NK cells in the high-risk group, which were associated with worse OS and PFS. This suggests that the IFN-γ response gene risk signature can predict tumor-infiltrating immune cells and direct the choice of clinical immunotherapies.

RBCK1 is essential for NF-κB stimulation and mutations in RBCK1 were associated with immunodeficiency (33–35). In the present study, the prognostic nomograms were constructed to predict the OS and PFS for 530 ccRCC patients, highlighting the significant prognostic implications of RBCK1 among the 24 IFN-γ response genes. RBCK1 was co-localized with tumor-infiltrating immune cells according to the three single-cell RNA datasets. This suggested that RBCK1 expression is correlated with tumor-infiltrating immune cells. The large-scale pan-cancer analysis also showed that RBCK1 had a strong prognostic value related to the significant differential expression of mRNAs between cancer and normal tissues and was correlated with tumor-infiltrating immune cells, tumor purity, and immune microenvironment scores. The RBCK1^high^ group had a significantly higher TIDE score compared with the RBCK1^low^ group in the ccRCC or RCC cohorts, which suggests a better response to the ICTs in RCC patients with RBCK1^high^ expression. In addition, studies in a real-world cohort (232 ccRCC patients from the FUSCC cohort based on proteomics sequencing data) showed that the immune-cold RBCK1^high^ group had a worse predicted prognosis than the RBCK1^low^ group. Inhibition of RBCK1 attenuates cell proliferation and migration and promotes cell apoptotic abilities of the 786-O cell. It is a limitation of the present study that only one cell line was used. After transfected with siRNA in human ccRCC cells, extraordinarily decreased cell proliferation, migration capacities, and prominently elevated apoptosis tumor cell proportion were found in the siRNA groups compared with the negative control group. In view of the findings of this study, future research directions can focus on the problem of RBCK1-regulated tumor microenvironment disorder mediating RCC malignant biological behavior. Furthermore, regarding the observed heterogeneity between the effect estimates in the two studies (TCGA and FUSCC), it is worth noting that these are retrospective studies, and the diagnostic criteria, ascertainment, and age distribution differ between them. On the other hand, we added the clinicopathological features of ccRCC patients and clarified that based on the retrospective study, IFN-γ response clusters and RBCK1 cannot yet be used as decision aids to spare patients from immunotherapy and/or clinical treatment management. These limitations will largely be addressed in a planned prospective multicenter research to confirm the benefits indicated from this study.

## Conclusion

In conclusion, this study investigated IFN-γ response clusters to derive a risk signature for papillary, chromophobia, and clear cell RCC patients, which can be used for determining a clinical diagnosis. These IFN-γ response signatures improved the prognostic accuracy of immune contexture in the ccRCC microenvironment. We performed a comprehensive analysis based on multi-omics large-scale data and found that immune-cold RBCK1^high^ patients had pro-tumorigenic immune infiltration and a significantly worse outcome compared with RBCK1^low^ patients. Our discovery of these novel independent prognostic indicators in RCC highlights the relationship between tumor phenotype and immune contexture.

## Data Availability Statement

The datasets presented in this study can be found in online repositories. The names of the repository/repositories and accession number(s) can be found below: https://www.ncbi.nlm.nih.gov/bioproject/PRJNA557348 and PRJNA557348.

## Ethics Statement

The studies involving human participants were reviewed and approved by the Ethics Committee of Fudan University Shanghai Cancer Center. The patients/participants provided their written informed consent to participate in this study. Written informed consent was obtained from the individual(s) for the publication of any potentially identifiable images or data included in this article.

## Author Contributions

Conceptualization: WX, WZ, JT, XT, CL, WL, and JS. Formal analysis: WX, WZ, WL, GW, and XT. Funding acquisition: WX, HZ, and DY. Investigation: WX, XT, WL, JT, AA, JS, and WZ. Resources: HH, GS, GW, CL, YQ, HZ, and DY. Supervision: YQ, HH, GW, CL, HZ, and DY. Original draft: WX, WZ, WL, and JT. Review and editing: GW, CL, YQ, HZ, and DY. All authors contributed to the article and approved the submitted version.

## Funding

This work was supported by grants from the National Key Research and Development Project (No. 2019YFC1316000); “Fuqing Scholar” Student Scientific Research Program of Shanghai Medical College, Fudan University (No. FQXZ202112B); Natural Science Foundation of China (No. 82172817); Natural Science Foundation of Shanghai (No. 20ZR1413100); and Shanghai Municipal Health Bureau (No. 2020CXJQ03).

## Conflict of Interest

The authors declare that the research was conducted in the absence of any commercial or financial relationships that could be construed as a potential conflict of interest.

The reviewer J-YZ declared a shared affiliation with several of the authors to the handling editor at the time of review. The reviewer HW declared a shared affiliation with several of the authors to the handling editor at the time of review.

## Publisher’s Note

All claims expressed in this article are solely those of the authors and do not necessarily represent those of their affiliated organizations, or those of the publisher, the editors and the reviewers. Any product that may be evaluated in this article, or claim that may be made by its manufacturer, is not guaranteed or endorsed by the publisher.
