# Supplementary figures and images for: Comprehensive Multi-Omics Identification of Interferon-γ Response Characteristics Reveals That RBCK1 Regulates the Immunosuppressive Microenvironment of Renal Cell Carcinoma
Source: Front Immunol. 2021 Nov 2;12:734646. doi: 10.3389/fimmu.2021.734646 (PMC8593147; doi:10.3389/fimmu.2021.734646)

CDKN1A

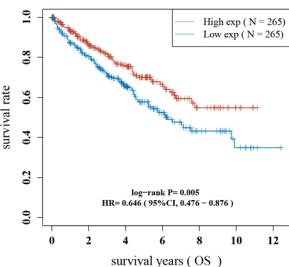

IRF8

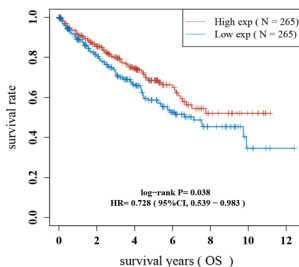

ITGB7

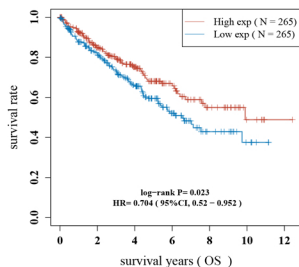

LAIS2

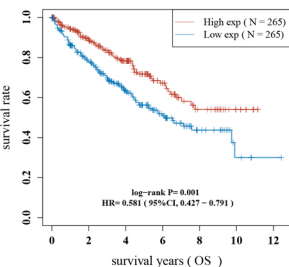

MT2A

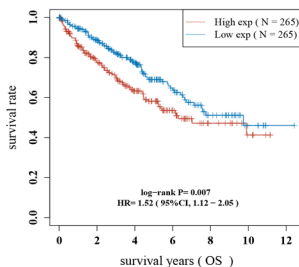

PFKP

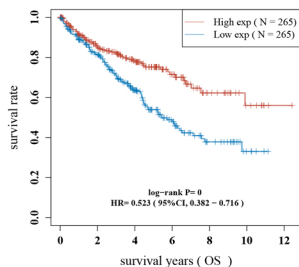

PNP

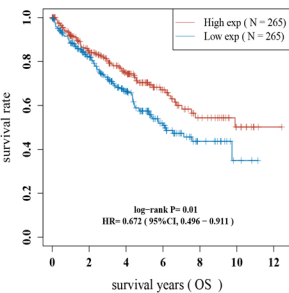

RBCK1

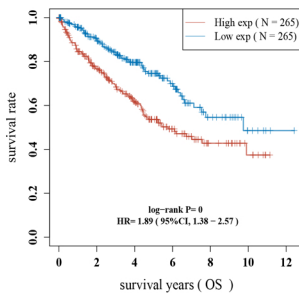

SELP

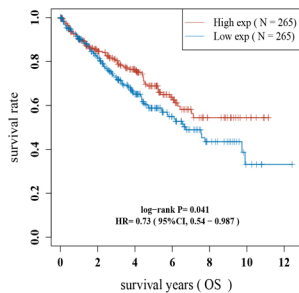

Supplement: Supplementary file 1 [file DataSheet_1.pdf]

**A**

# Overall survival

**TRAFD1**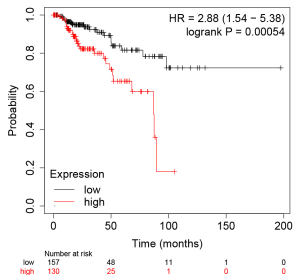**SOD2**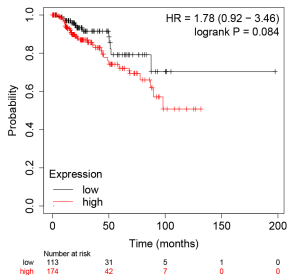**RIPK2**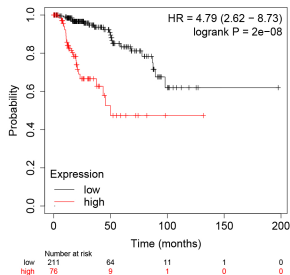**RBCK1**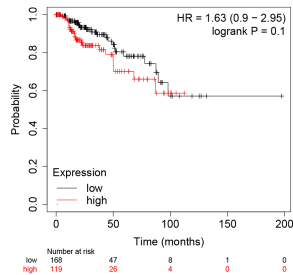**MT2A**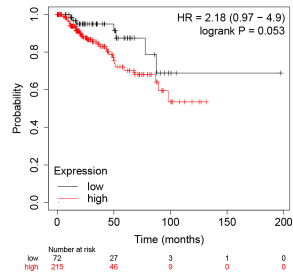**B**

# Progression-free survival

**TRAFD1**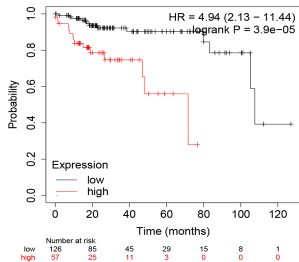**SOD2**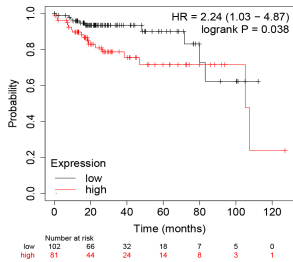**RIPK2**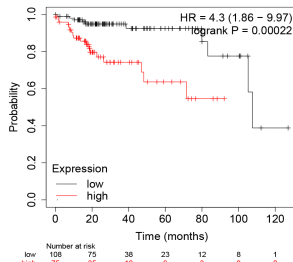**RBCK1**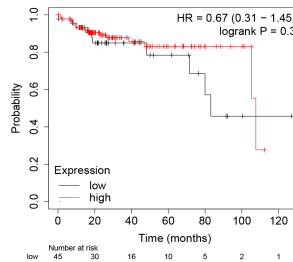**MT2A**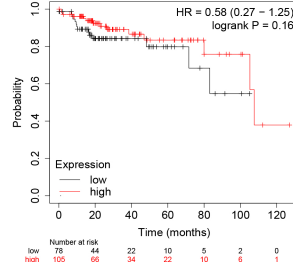

Supplement: Supplementary file 2 [file DataSheet_2.pdf]

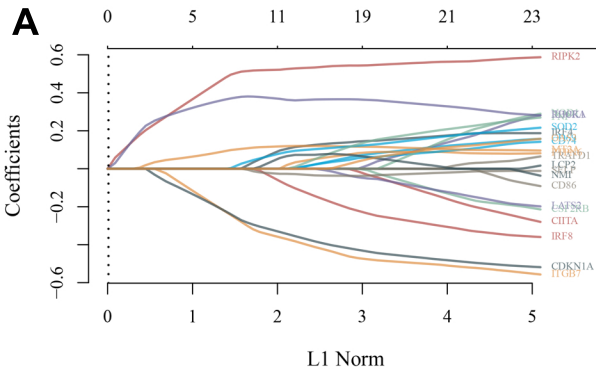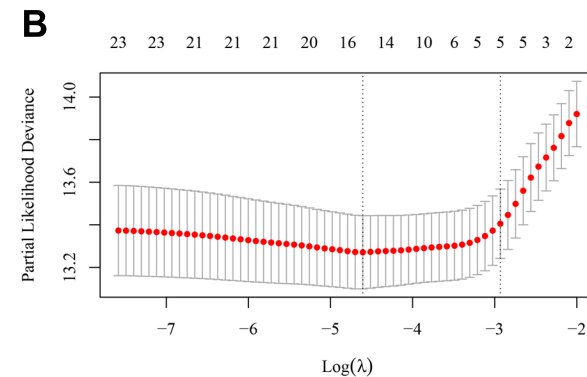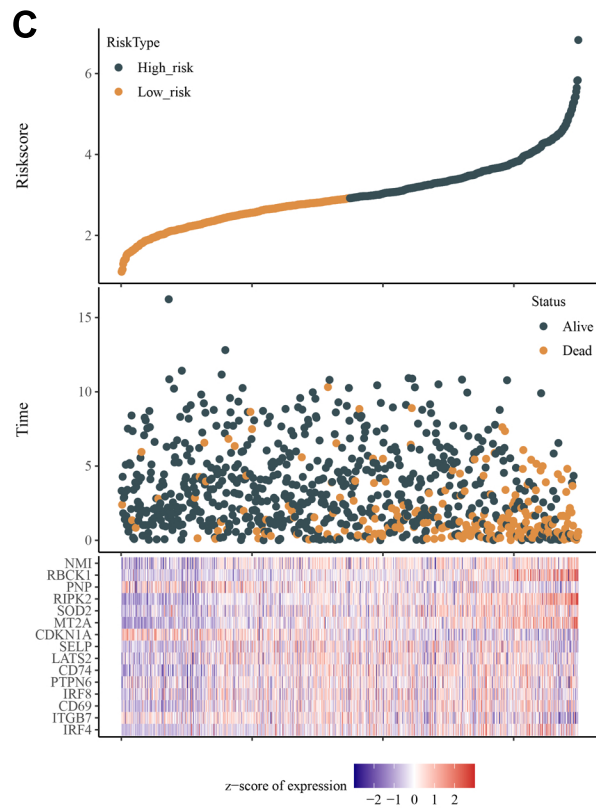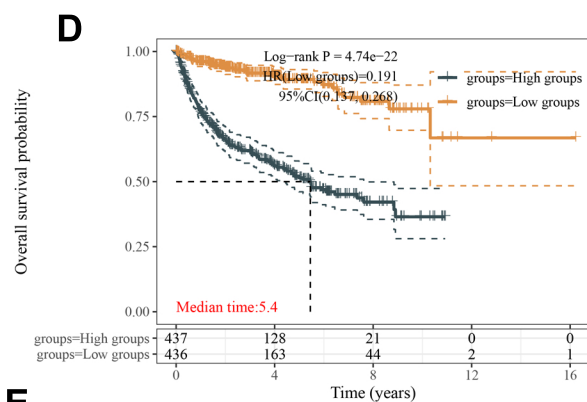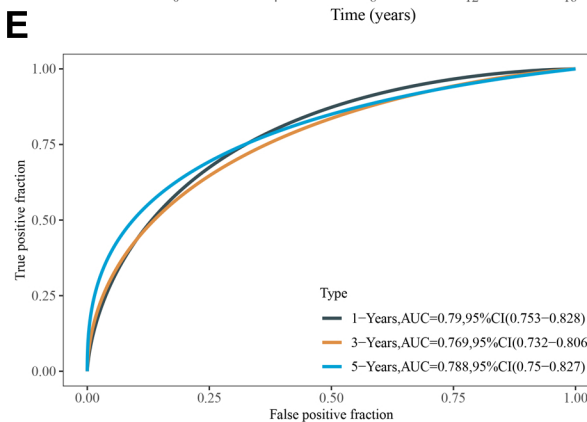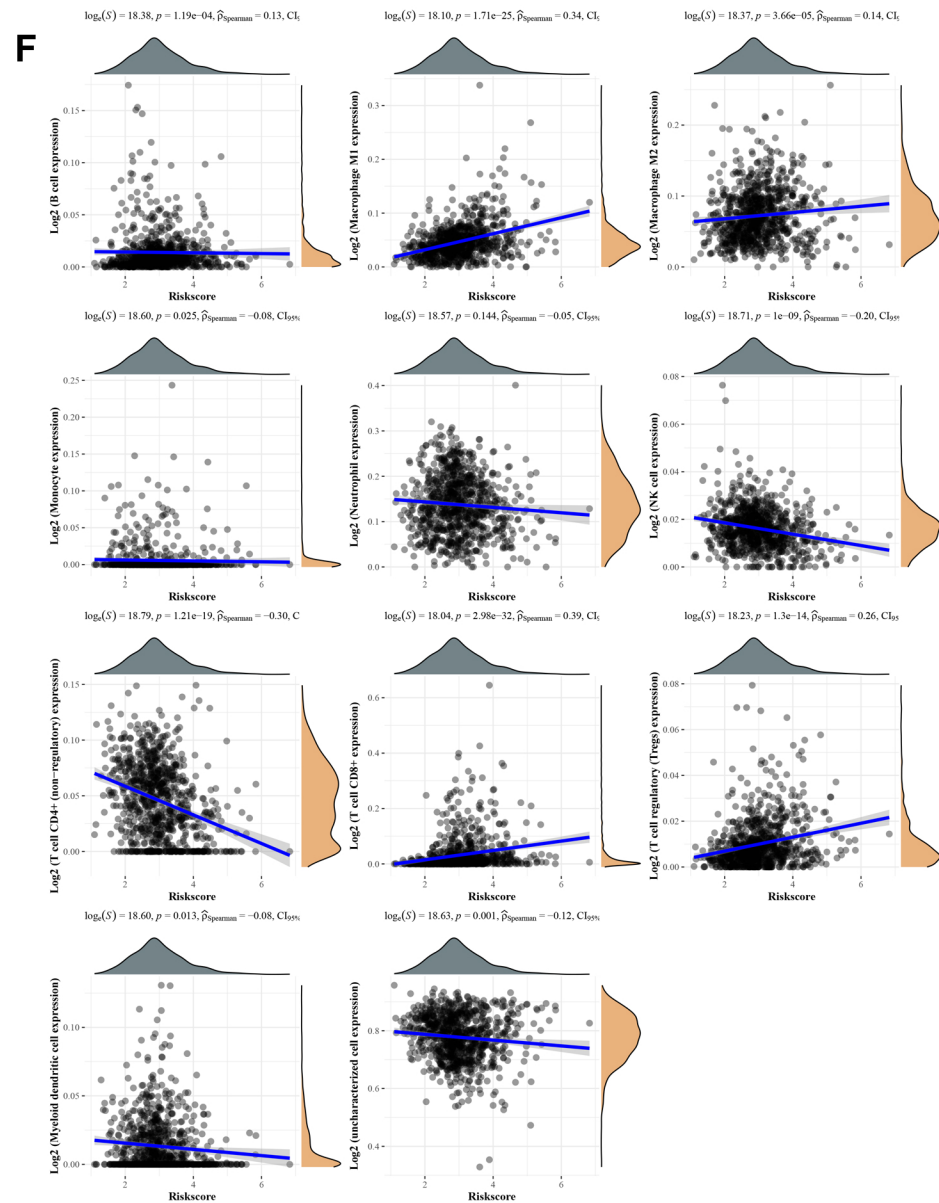

Supplement: Supplementary file 3 [file DataSheet_3.pdf]

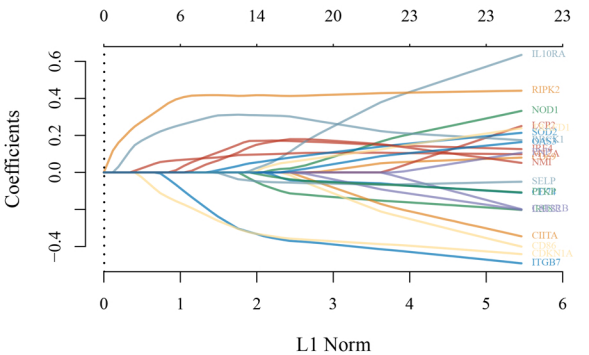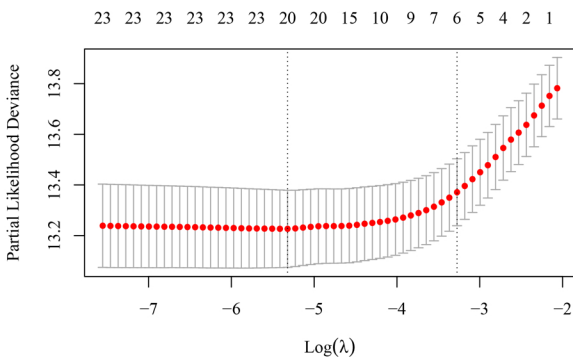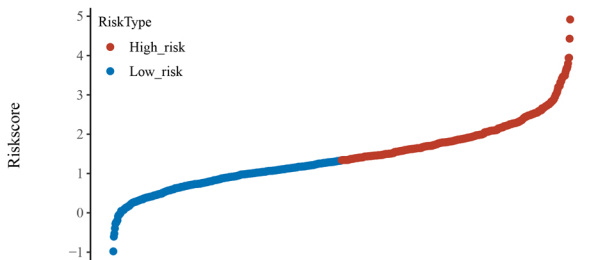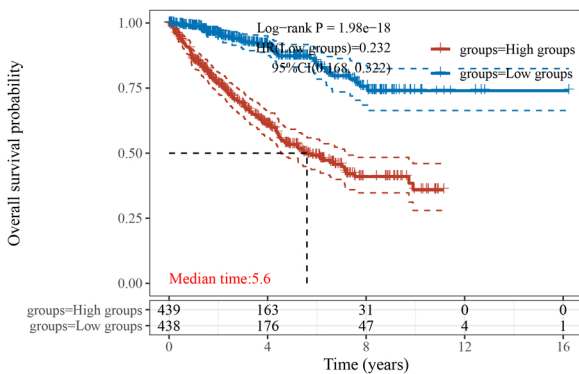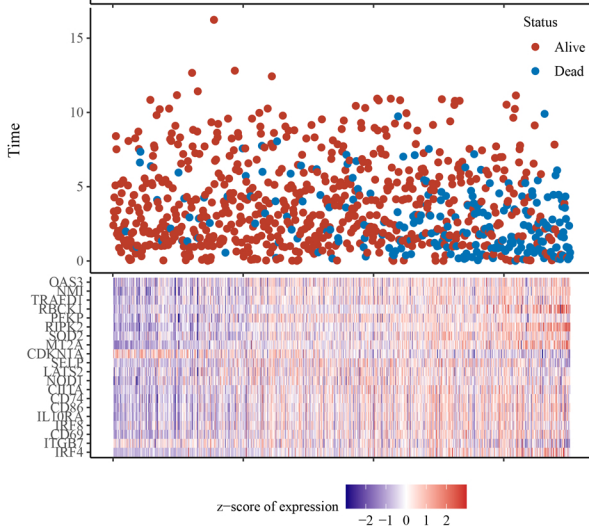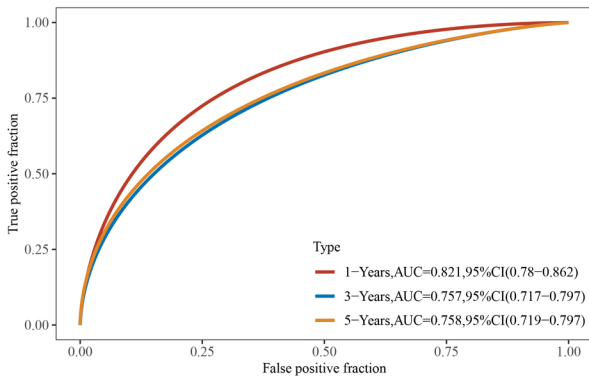

Supplement: Supplementary file 4 [file DataSheet_4.pdf]

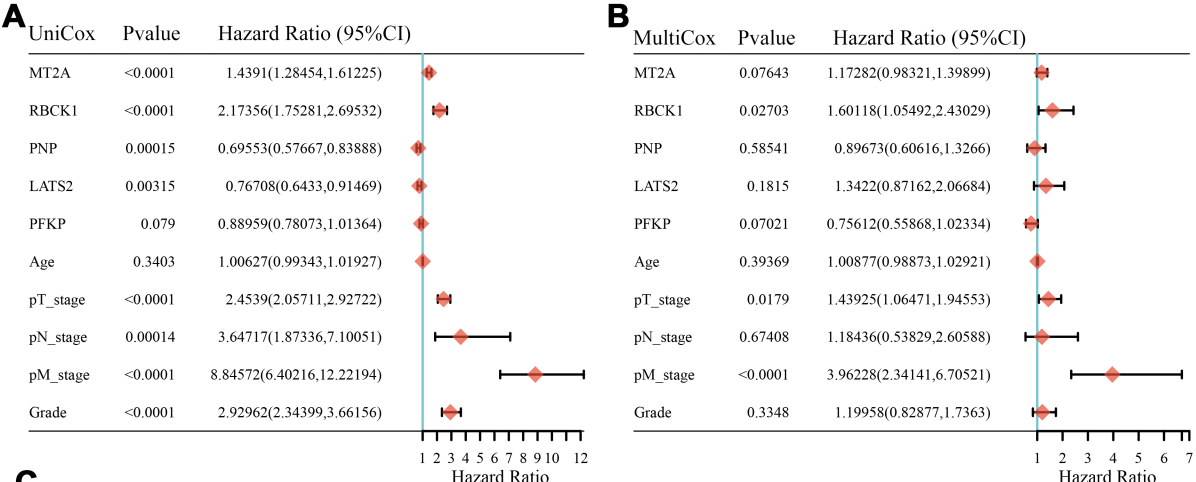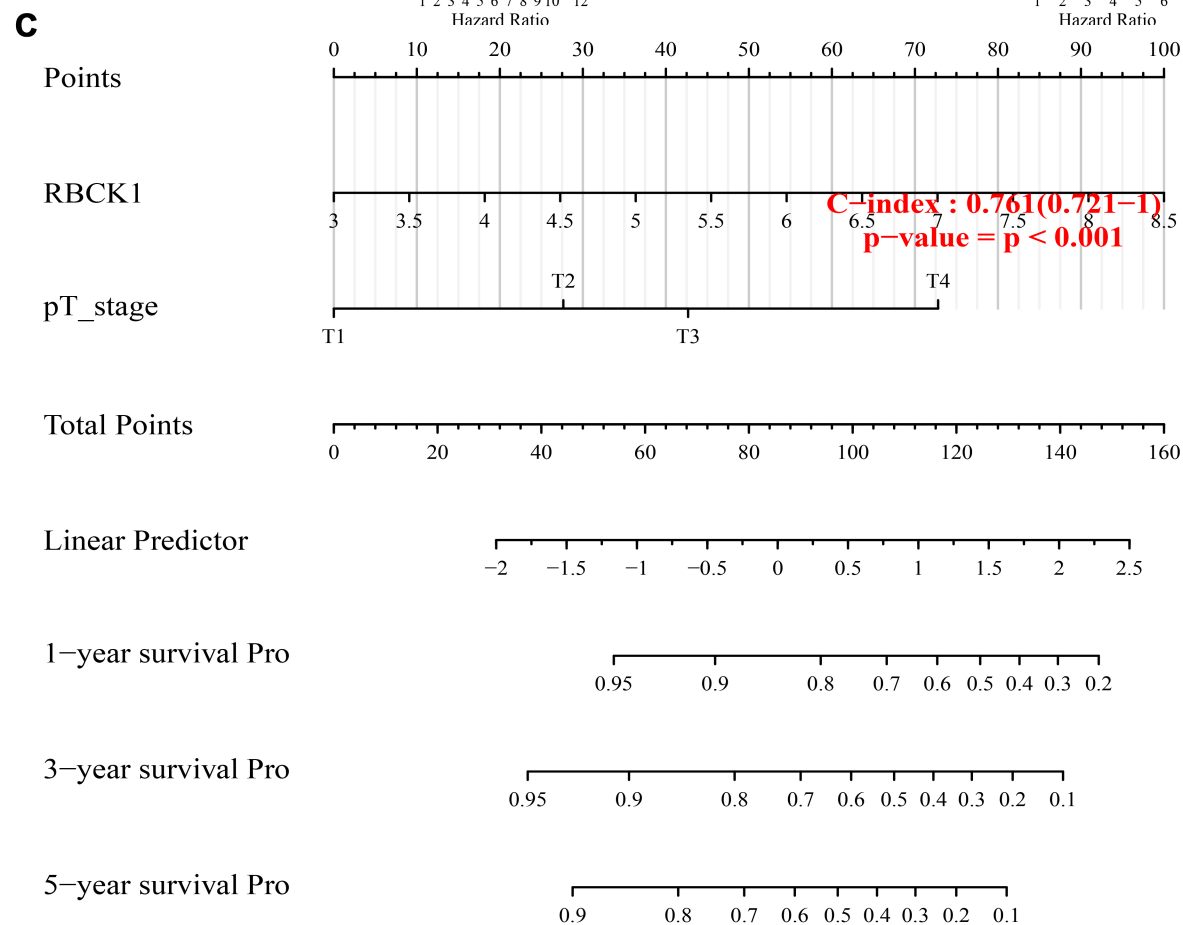

Supplement: Supplementary file 5 [file DataSheet_5.pdf]

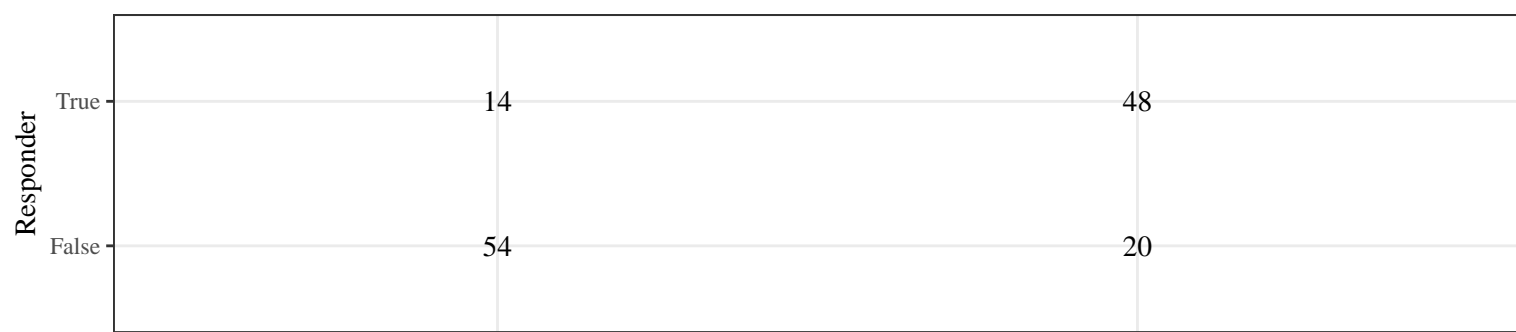

wilcox.tests p=2.5e-08

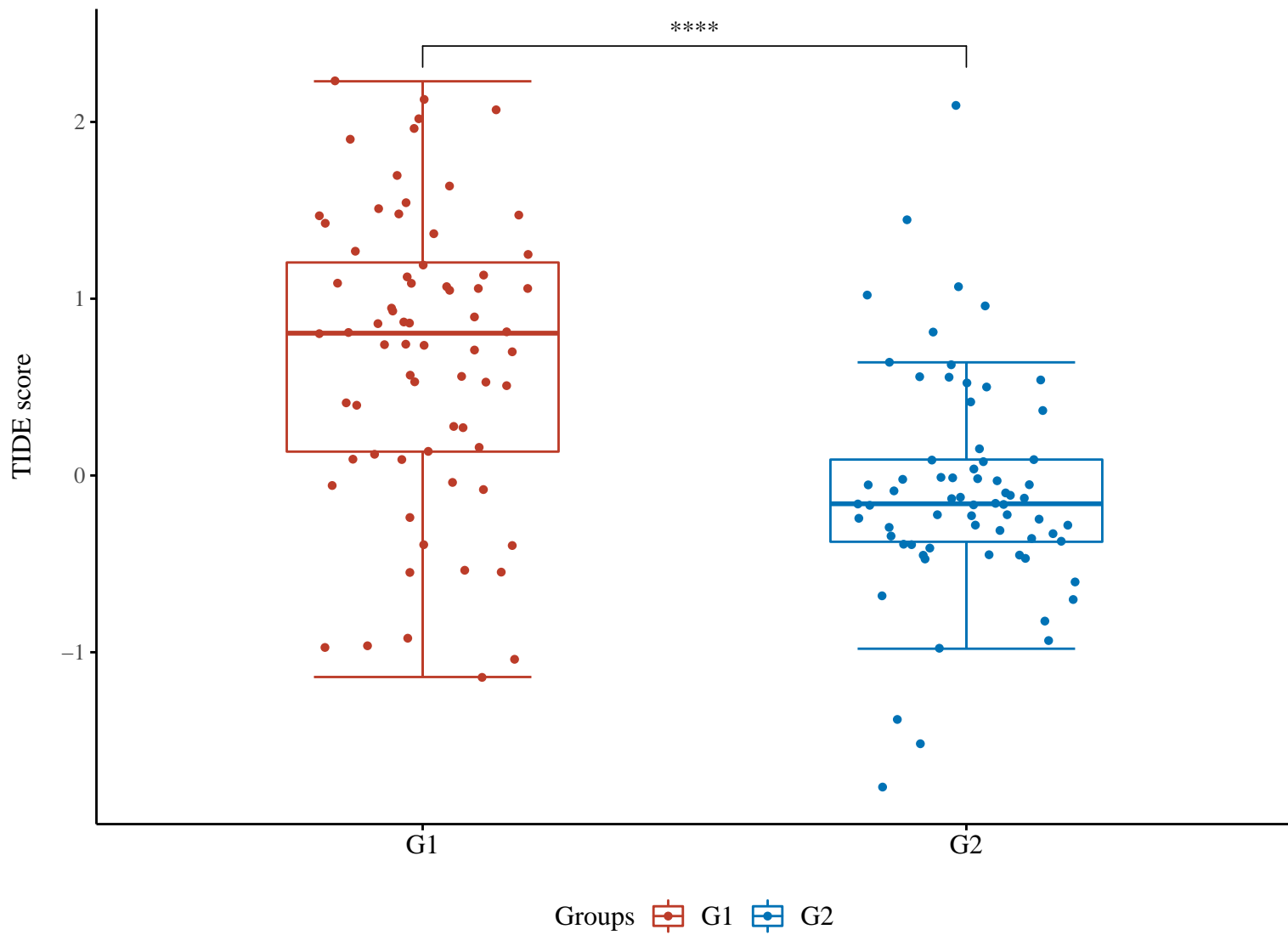

Supplement: Supplementary file 6 [file DataSheet_6.pdf]
